# Factors Associated with the 4-Year Estimated Incidence of Type 2 Diabetes Mellitus by Sex in Korean Adults: Secondary Data Analysis

**DOI:** 10.1155/2021/8547950

**Published:** 2021-12-06

**Authors:** KyungAe Kim, MiRan Bang

**Affiliations:** Kyungdong University, College of Nursing, 815, Gyeonhwon-ro, Wonju 26495, Republic of Korea

## Abstract

It is important to prevent the increase in the prevalence of diabetes mellitus (DM) worldwide by efficiently managing its controllable risk factors. This study aimed to identify factors associated with the 4-year estimated incidence of type 2 DM (T2DM) by sex and provide basic data for a sex-specific strategic approach to lifestyle modification. We performed a secondary data analysis using raw data from the seventh Korea National Health and Nutrition Examination Survey (KNHANES, 2016–2018). The KNHANES is a descriptive correlational survey designed to examine sex differences in the factors associated with the 4-year estimated incidence of T2DM. This study included 9,614 Korean adults (4,134 men and 5,480 women) aged 40–69 years without a diagnosis of T2DM. For the statistical analysis, a complex sample analysis was performed for sex comparison using the *χ*^2^-test or one-way analysis of variance; a multiple regression analysis was performed to analyze the sex-specific influencing variables of 4-year estimated T2DM incidence. The waist-to-height ratio, an indicator of central obesity in adults, showed the strongest association with the 4-year estimated incidence of T2DM in both groups (male: *β* = 0.33, *p* ≤ 0.001; female: *β* = 0.38, *p* ≤ 0.001). The influencing variables were the monthly drinking rate (*β* = 0.07, *p* ≤ 0.001) and sleep time (*β* = −0.03, *p* < 0.05) in men and the sedentary time in women (*β* = 0.03, *p* < 0.05). The overall explanatory power of these variables was 11.3% for men and 14.3% for women. Thus, significant sex differences were found in the 4-year estimated incidence of T2DM. Therefore, intervention programs need to be sex-specific to enhance the efficacy of the interventions in reducing the incidence of T2DM, and such intervention programs should be administered with a strategic approach differentiated by sex.

## 1. Introduction 

Worldwide, the number of adult patients with diabetes mellitus (DM) nearly doubled between 2006 (246 million) and 2019 (463 million). Especially, approximately 4 million adults aged 20–79 years have died from DM, with those aged ≤60 years accounting for 46.1% [1]. However, the age at DM diagnosis is gradually decreasing in Korea [2], with a significant sex difference in prevalence (i.e., 11.8% vs. 15.9% in women and men, respectively) [3]. The continuously increasing prevalence of DM and rising healthcare costs, along with a plethora of DM-induced complications [4], highlight the importance of preventing DM before its onset. However, the main symptoms of type 2 DM (T2DM), which include polydipsia, polyuria, and weight loss, are slight initially, and the patient is often unaware of this disease. Therefore, T2DM remains undiagnosed in one-third of such patients until a complication develops [5]. Therefore, it is crucial to reduce the incidence of DM by identifying the factors associated with T2DM.

The risk factors for T2DM include abdominal obesity, obesity, cardiovascular disease, family history of DM, previous history of impaired fasting glucose or impaired glucose tolerance, gestational DM, hypertension, insulin resistance, and alcohol consumption [3, 5–8]. In Korea, the obesity rate for men is 1.5 times higher than that for women [9]; however, the incidence of chronic diseases in women is increasing because of age-associated increases in abdominal obesity [10]. This is important, as 54% of T2DM cases are associated with abdominal obesity [3]. Alcohol consumption is an important risk factor among the Korean adult population, with 75% of men drinking at least once a month and alcohol consumption steadily rising among women over the past 10 years [9]. Drinking is associated with sleep disorders and poor sleep quality, which were induced by drinking, and affects men more than women [11]. Sleep deprivation has been reported to increase the risk of T2DM in some studies [12, 13]; in contrast, no association between sleep duration and DM was observed in another previous work [14].

Therefore, it may be assumed that risk factors and the onset of T2DM may be sex independent (e.g., genetic factors and lifestyle factors) or sex specific because of pregnancy and hormonal changes [6]. To prevent the onset of T2DM, particular care should be taken in the prediabetic stage to systematically improve lifestyle, reduce weight, and avoid an accumulation of excess body fat in the abdomen [7]. Additionally, moderate-intensity physical activity, reduction of sedentary time, and drug therapy could help prevent the onset of T2DM [5, 15]. In this regard, physical activity decreased in the Korean adult population and a sex difference was also observed in physical activity practices [16]. Moreover, given the steady increase in alcohol consumption among women, it is necessary to accurately identify factors associated with T2DM's sex-specific incidence.

This study aimed to examine the effects of sex-specific lifestyle factors associated with the 4-year estimated incidence of T2DM in the Korean adult population. These data would support the development of educational materials and customized intervention programs for T2DM prevention.

## 2. Materials and Methods

### 2.1. Study Design

This study is a secondary analysis using data from the Seventh Korea National Health and Nutrition Examination Survey (KNHANES VII) (2016–2018). KNHANES is a descriptive correlational survey designed to examine sex differences in the factors associated with the 4-year estimated incidence of T2DM.

### 2.2. Participants

**Figure 1:**
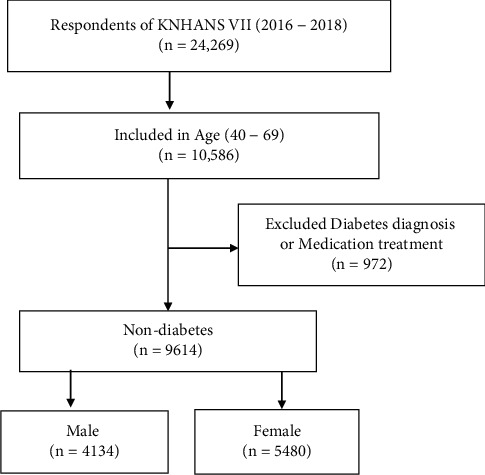
Flowchart of the inclusion of the study population.

The data for this study were extracted from the raw data of KNHANES VII using a two-stage stratified cluster sampling scheme. The primary and secondary sampling units were 13,248 households in 576 enumeration districts across the country. The sampling frames were stratified according to si-do (municipalities and provinces), dong-eup-myeon (local administrative districts), and housing type (single-family housing, multifamily housing); the floor area and education ratios were the endogenous stratification criteria. In complex sample data, the number of participants varied because of missing values on each variable. Thus, among the total respondents of the KNHANES VII (*n* = 24,269), those aged 40–69 years (*n* = 10,586) were extracted as the target population of this study. As the middle age range was expanded and defined as 40–69 years in the model for predicting the risk of T2DM in Korean patients [17], those with ages within this range were primarily considered as the target population. After excluding 972 patients diagnosed with or treated for DM, 9,614 patients (4,134 men and 5,480 women) were selected for the analysis (Figure 1).

### 2.3. Study Variables

#### 2.3.1. General Characteristics

The sociodemographic characteristics of the participants (age, sex, household composition, education level, household income level, occupation, and perceived health status) were investigated.

Body mass index (BMI, kg/m^2^) was classified into four categories according to the World Health Organization criteria: <23 (normal weight), 23–24.9 (overweight), 25–29.9 (preobesity), and ≥30 kg/m^2^ (obesity) [17, 18].

Blood samples were collected after at least 8 h of fasting. The levels of total cholesterol, triglycerides, high-density lipoprotein (HDL) cholesterol, and low-density lipoprotein cholesterol were measured.

The amount of physical activity of each participant was calculated based on their response to the International Physical Activity Questionnaire (IPAQ), using the formula for calculating the metabolic equivalent task minutes per week (MET-min/week) after summing the duration (minutes) of each physical activity [19]. The amount of physical activity, which was expressed in MET-min/week, was classified into four intensity categories based on the reference value of 600 MET-min/week, in accordance with the minimum weekly amount of physical activity recommended by the American Diabetes Association and American Heart Association [6, 20].

#### 2.3.2. Central Obesity

Central obesity was measured using the waist-to-height ratio (WHtR) based on the circumference of the waist and height measured by the KNHANES survey team. It was classified based on the cutoff value of WHtR (0.53) for cardiovascular risk and metabolic disease in Asians [21].

#### 2.3.3. Sedentary Time

Sedentary time was defined as the time spent sitting or lying, excluding the sleeping time while working, staying at home, and socializing. In a previous study, the sedentary reference time associated with T2DM incidence was found to be 8 h per day [22].

#### 2.3.4. Monthly Drinking Rate

The monthly drinking rate, defined as the frequency of drinking at least once a month within the past 12 months, was calculated. The total number of participants was the denominator, while the number of participants who reported that they drank alcohol at least once a month over the last year was considered the numerator, using available original data. The respondents were categorized based on the quantity consumed into two groups: 0–1 and ≥1 glass per month categories, regardless of the type of alcoholic beverage [23].

#### 2.3.5. Sleep Time per Day

Sleep time, defined as the daily duration of sleep expressed in minutes, was obtained by dividing the weekly total number of hours of night's sleep by seven. The reference daily average sleep time for good sleep quality was set at 7–8 h and sleep times <7 or >8 h indicated poor sleep quality [24].

#### 2.3.6. The 4-Year Estimated Incidence of T2DM

The 4-year estimated incidence of T2DM was obtained using a risk prediction model based on clinical variables (e.g., age, parental or sibling history of DM, smoking status, BMI, and hypertension) and biomarkers (e.g., fasting plasma glucose, HDL cholesterol, triglyceride, and glycated hemoglobin [HbA_1c_]). In this model, the 4-year estimated incidence of T2DM was calculated in two steps. In Step 1, each variable was categorized, and each category was substituted with a score calculated using the calculation formula. Then, in Step 2, the final score was obtained by substituting the raw score with the adjusted score [17].

### 2.4. Data Collection

The data used in this study were drawn from the KNHANES VII, a national health and nutrition survey conducted by the Korea Disease Control and Prevention Agency (KDCA) from January (2016) to December (2018). The participants selected by a stratified cluster sampling scheme underwent a health behavior survey and examination by a professional survey team using a mobile examination center.

The health behavior survey was conducted using an interview for sociodemographic items (e.g., education and occupation) and a self-report questionnaire for health behavior (e.g., smoking and drinking). The examination was conducted by direct measurement, observation, and sample analysis. To ensure accurate examination results, the investigators were trained on examination skills and tested in a regular training program of seven sessions.

### 2.5. Ethical Considerations

The raw data from the KNHANES VII are anonymized according to the Personal Information Protection Act and the Statistics Act. The survey was conducted with the approval of the KDCA (approval no. 2018-01-03-P-A). The researchers downloaded the datasets and data codebook in compliance with the regulations on the KDCA raw data disclosure procedure on the KNHANES website (https://knhanes.cdc.go.kr/). The institutional review board of the affiliated university approved the secondary data analysis survey (approval no. 1041455-202004-HR-002-01).

### 2.6. Data Analysis

Data analysis was performed using the IBM SPSS Statistics for Windows, version 21.0 (IBM Corp., Armonk, NY, USA). The KNHANES used two-stage stratified cluster sampling to collect data and applied stratified clustering and weighting schemes, which were reflected in the complex sample analysis study design. Missing values were analyzed as significant values in each variable. Descriptive statistics were used to evaluate the participants' general characteristics, variables for the risk scores of the sex-specific 4-year estimated incidence of T2DM, and major risk factors for DM. Tolerance and the variance inflation factor (VIF) were examined to check for multicollinearity between independent variables. Multiple linear regression analysis was performed to check for sex differences in the factors associated with the 4-year estimated incidence of T2DM and determine the explanatory power of each factor. There was no multicollinearity problem in the regression model with tolerance and VIF values of <1 and <10, respectively [25]. Factors affecting the prediction rate of DM occurrence after 4 years were presented only after removing insignificant variables through hierarchical regression analysis. A *p*-value of <0.05 was considered statistically significant.

## 3. Results

### 3.1. Participants' General Characteristics

The rate of one-person households due to divorce, widowhood, separation, or family members moving out showed a significant sex difference. Men and women living alone accounted for 8.9% and 7.1% of the study population, respectively (*x*^2^ = 6.54, *p*=0.011). Sex differences were observed in the education level (*x*^2^ = 129.18, *p* ≤ 0.001), household income level (*x*^2^ = 36.16, *p* ≤ 0.001), occupation (*x*^2^ = 181.13, *p* ≤ 0.001), and perceived health status (*x*^2^ = 42.96, *p* ≤ 0.001) (Table 1).

### 3.2. Four-Year Sex-Specific Estimated Incidence of T2DM

A significant sex difference was observed in the variables associated with the 4-year estimated incidence of T2DM. Concerning the risk scores, a significant sex difference was observed in the variable “family history of DM” (*x*^2^ = 21.82, *p* ≤ 0.001). Additionally, a significant sex difference was observed in the BMI (*t* = 106.59, *p* ≤ 0.001), HDL cholesterol level (*t* = 823.91, *p* ≤ 0.001), and estimated score for T2DM incidence after 4 years (*x*^2^ = 207.29, *p* ≤ 0.001) (Table 2).

### 3.3. Major Risk Factors for the 4-Year Sex-Specific Estimated Incidence of T2DM

On examining the sex differences in risk factors for the 4-year estimated incidence of T2DM in the Korean adult population, significant sex differences were observed in the WHtR (*t* = 13.75, *p* ≤ 0.001) and daily mean sleep time (*t* = 4.07, *p*=0.044). Moreover, significant sex differences were observed in the sedentary time (*t* = 10.70, *p* ≤ 0.001) and monthly drinking rate (*t* = 733.33, *p* ≤ 0.001) (Table 3).

### 3.4. Factors Associated with the 4-Year Estimated Incidence of T2DM according to Sex

Multiple linear regression analysis was conducted to identify sex differences in the factors associated with the 4-year estimated incidence of T2DM in the Korean adult population. The significant risk factors associated with the 4-year estimated incidence of T2DM were the WHtR (*β* = 0.33, *p* ≤ 0.001), monthly drinking rate (*β* = 0.07, *p* ≤ 0.001), and sleep time (*β* = −0.03, *p* < 0.05) in men, with an overall explanatory power of 11.3%; the WHtR (*β* = 0.38, *p* ≤ 0.001) and the sedentary time (*β* = 0.03, *p* < 0.05) were found to be the significant risk factors in women, with an overall explanatory power of 14.3% (Table 4).

## 4. Discussion

This study verified whether there are sex differences in the factors associated with the 4-year estimated incidence of T2DM in the Korean adult population in relation to their lifestyles. This finding is of great importance as it provides the rationale that supports a sex-specific approach to reduce the incidence of DM. Moreover, our study provides basic data for developing sex-specific intervention programs to prevent the onset of DM.

Among the factors associated with the 4-year estimated incidence of T2DM by sex, the most influential factor in both men and women was the WHtR, an indicator of central obesity. This finding supported the results of an 8-year cohort study in which 9,753 men and 15,491 women aged 35–65 years who participated in the European Prospective Investigation into Cancer and Nutrition-Potsdam were followed up, to determine the risk factors for the incidence of T2DM. In this work, the investigators found that normal-weight participants (BMI <25 kg/m^2^) with a large waist circumference had a higher relative risk (male: 3.62; female: 2.74) than overweight patients (BMI: 25–29.9 kg/m^2^) with a small waist circumference (male: 2.26; female: 1.40) [26]. This was also consistent with the findings of a study in which the incidence rates of DM in women were 26% and 74% in the WHtR 0.51–0.6 and WHtR ≥ 0.6 groups, respectively, demonstrating that abdominal obesity was a major risk factor for the incidence of DM [27]. Furthermore, there was a significant sex difference in the WHtR and BMI in this study with 33.1% vs. 32.5% and 39.0% vs. 25.2% men and women having WHtR ≥ 0.53 and BMI 25–29.9 kg/m^2^, respectively. This finding highlighted the importance of identifying the cause of obesity in men and promoting and implementing active lifestyle changes and interventions, respectively, to maintain a normal weight and healthy waist circumference.

The variable “sedentary time” had a significant effect on the 4-year estimated incidence of T2DM in women but not in men. The results regarding the sedentary time in women were consistent with the findings of a study in which 655 out of 5,829 participants (54% men and 46% women) developed T2DM during a follow-up period of 11.1 years. Physical activity reduced the incidence of T2DM, and the incidence of T2DM increased with an increase in leisure sedentary time (1.65-fold) and television watching (2.68-fold) [28]. In contrast, in a cohort study of 712 adults, moderate-to-vigorous-intensity physical activity was identified as an important indicator of the incidence of DM; however, sedentary time showed no association with mortality and incidence of DM [29]. This was consistent with our results, showing that the sedentary time was not a significant predictor of T2DM in men.

This difference in research results could be attributed to the fact that increases in physical activities reduce the sedentary time [15]. In this study, there was a significant difference between men and women in physical activity per week. For example, 23.7% of men and 19.0% of women who practiced >1,500 MET exercises showed low physical activity in both groups. Thus, it can be inferred that men with more physical activities than women have relatively less sedentary time, which reduces the incidence of DM. However, it is necessary to conduct a longitudinal study on the prevalence of DM according to the sedentary time and pattern in men and women. An important implication of this study is the need to develop appropriate intervention programs that can help high-risk groups live an active life to prevent the incidence of T2DM.

Among the lifestyle factors, alcohol consumption was significantly associated with the 4-year estimated incidence of T2DM in men but not in women. This result was partially consistent with that of another study indicating that the quantity and frequency of alcohol consumption were associated with the incidence of DM. These results were observed after following 28,704 men and 41,847 women for 4.6 years, during which 859 men and 847 women developed DM [30]. In this study, the significant association between drinking and DM, which was observed only in men, may be attributable to Korean men's negative lifestyle habits of resorting to alcohol, tobacco, and drugs, instead of positively coping with their emotional vulnerabilities and stress under the pressure of their responsibilities as employees and breadwinners [31]. Moreover, we found that the Korean men's monthly drinking rate was 1.7-fold higher than that of female participants (74.3% vs. 43.5%). This highlights the need for active intervention to reduce the quantity and frequency of alcohol consumption in men. Furthermore, given the Diabetes Fact Sheet report in Korea [3], 23.1% of patients with DM are heavy drinkers; therefore, there is an urgent need to reduce alcohol consumption. Additionally, with the recent increase in women's alcohol consumption [9], which exposes them to increased risk of DM development, it is necessary to promote educational and promotional measures to raise awareness that the quantity and frequency of alcohol consumption are risk factors for developing T2DM.

The significant difference in sleep time between men and women, with an association between sleep time and the incidence of DM only in men, which were observed in our study, was consistent with the results of a previous study involving 16,893 Chinese adults. The study revealed that the incidence of DM increased in the groups with <6 (1.4-fold) and >8 h (1.39-fold) of sleep, compared with the group with 6–8 h of sleep [24]. This alcohol-related sex difference in sleep quality was in line with the findings of a study investigating the association between quantity of alcohol consumption and the quality of sleep, which showed that men were likely to suffer more than women from poor sleep quality after drinking [32]. Thus, our results highlighted sex differences among the participants. Considering that drinking reduces sleep quality, causes sleep disturbances, and affects daily activities [33], excessive drinking should be controlled to improve sleep quality and quantity. However, with the drinking rate of men remaining at a high level every year, there is an urgent need to identify intervention measures to reduce alcohol consumption effectively.

This study had several limitations. First, the health behavior survey was based on a self-reported questionnaire, which relied on the individual's memory that may differ from reality. Additionally, the limited information derived from the questionnaire items will have to be complemented by follow-up research with a longer study period and different methodologies. Second, the KNHANES is a cross-sectional survey that focuses on understanding the intervariable relationships rather than explaining their causalities and does not lend itself well to determining the causal relationship between the 4-year estimated incidence of T2DM and each of the lifestyle habits investigated. Therefore, a longitudinal study is required to determine the effects of long-term, lifestyle-improving intervention programs in reducing the prevalence of DM. Despite these limitations, our findings would be of great importance for the readership, as we showed significant sex differences in factors associated with the 4-year estimated incidence rates of T2DM. Interestingly, the obtained results were different from those of other studies that focused on identifying the risk factors for DM. This study highlights the need to adopt sex-specific approaches to reduce the incidence of DM. These approaches are expected to serve as important data for designing appropriate intervention programs.

## 5. Conclusion

In this study, the 4-year estimated incidence of T2DM was calculated for normal adults aged 40–64 years, and the factors affecting the predicted 4-year incidence rate of T2DM according to sex were identified.

Central obesity was most strongly associated with the 4-year estimated incidence of T2DM in both sexes. Other factors observed to have a negative effect on the incidence of T2DM were the quantity and frequency of alcohol consumption, sleep duration shorter or longer than 7–8 h in men, and sedentary time in women.

As observed in this study, these sex differences in the incidence of T2DM highlighted the need to adopt sex-specific approaches and lifestyle-improving intervention programs. These results would help implement active sex-specific strategies for educational and promotional interventions. The results of this study can be used as basic data for exploring strategic measures to reduce the incidence of T2DM and to develop intervention programs that apply strategies to enhance compliance. Furthermore, it is necessary to follow the effects of such programs in a longitudinal study.

## Figures and Tables

**Table 1:** Comparison of the participants' general characteristics according to sex (*n* = 9,614).

Variables	Male (*n* = 4,134)	Female (*n* = 5,480)	*t* or *x²*	*p* value
	*n* (%) or M ± SE	*n* (%) or M ± SE		
Age (years)	52.39 ± 0.15	52.81 ± 0.15	7.20	0.008
Living with				
Spouse or children	3,748 (91.1)	5,005 (92.9)	6.54	0.011
Living alone	386 (8.9)	475 (7.1)		
Education				
≤Middle school	1,330 (25.6)	2,085 (32.9)	129.18	≤0.001^*∗*^
High school	1,223 (31.6)	1,853 (37.4)		
≥College	1,581 (42.8)	1,542 (29.6)		
Household income (10,000 won/month)				
≤100	455 (9.8)	788 (13.1)	36.16	≤0.001
101–300	2,181 (52.7)	2,915 (53.7)		
≥300	1,498 (37.5)	1,777 (33.2)		
Occupation				
Managerial/professional	1,194 (32.5)	1,002 (19.1)	181.13	≤0.001
Service/sales	360 (10.0)	997 (19.4)		
Routine/manual	1,508 (37.7)	995 (17.6)		
Unemployed/housewife	1,072 (19.7)	2,486 (43.8)		
Perceived health status				
Good	1,169 (30.5)	1,324 (25.0)	42.96	≤0.001
Moderate	2,457 (56.9)	3,196 (57.5)		
Poor	508 (12.6)	960 (17.5)		

^*∗*^ *p* < 0.05; *n*: unweighted count; %: weighted count; SE: standard error; M: mean.

**Table 2:** Comparison of the 4-year estimated incidence of T2DM according to sex (*n* = 9,614).

Variables	Male (*n* = 4,134)	Female (*n* = 5,480)	*t* or *x²*	*p*-value
	*n* (%) or M ± SE	*n* (%) or M ± SE		
Age (years)				
40–44	762 (20.1)	958 (18.7)	8.18	0.004
45–49	757 (21.5)	989 (20.8)		
50–54	688 (19.0)	920 (18.6)		
55–59	735 (18.1)	998 (18.5)		
60–64	633 (12.7)	875 (13.9)		
65–69	559 (8.6)	740 (9.5)		
Family history of diabetes				
No	3,259 (76.6)	4,025 (71.8)	21.82	≤0.001^*∗*^
Yes	875 (23.4)	1,455 (28.2)		
Currently smoking				
No	2,476 (62.2)	5,030 (94.6)	1,077.28	≤0.001
Yes	1,426 (37.8)	260 (5.4)		
BMI (kg/m^2^)	24.55 ± 0.05	23.69 ± 0.06	106.59	≤0.001
<23	1,130 (29.6)	2,374 (47.0)	110.24	≤0.001
23–24.9	1,036 (27.0)	1,169 (22.7)		
25–29.9	1,483 (39.0)	1,338 (25.2)		
≥30	158 (4.4)	270 (5.1)		
Hypertension				
No	3,236 (78.4)	4,534 (84.3)	43.48	≤0.001
Yes	898 (21.6)	946 (15.7)		
FPG (mg/dL)	102.48 ± 0.41	96.64 ± 0.25	146.10	≤0.001
<90	608 (16.2)	1,440 (28.8)	261.44	≤0.001
90–99	1,402 (36.9)	2,183 (42.7)		
≥100	1,810 (46.9)	1,502 (28.5)		
High-density lipoprotein cholesterol (mg/dL)	47.06 ± 0.21	54.84 ± 0.21	823.91	≤0.001
<35	457 (11.9)	164 (3.1)	567.74	≤0.001
35–49	1,952 (52.1)	1,665 (32.1)		
≥50	1,366 (36.0)	3,215 (64.8)		
Triglyceride (mg/dL)	181.64 ± 3.60	118.47 ± 1.36	271.90	≤0.001
<120	1,561 (40.0)	3,259 (64.5)	560.40	≤0.001
120–149	586 (15.4)	721 (14.1)		
≥150	1,673 (44.6)	1,145 (21.4)		
HbA_1c_ (%)	5.65 ± 0.01	5.58 ± 0.01	20.30	≤0.001
<5.5 (37 mmol/mol)	1,884 (53.3)	2,561 (54.4)	0.80	0.371
5.5–6.4 (37–46 mmol/mol)	1,722 (46.7)	2,375 (45.6)		
Estimated score of diabetes incidence after 4 years (%)	11.55 ± 0.19	7.87 ± 0.13	285.08	≤0.001
<11	2,384 (55.7)	4,103 (74.8)	207.29	≤0.001
11–20	1,108 (27.9)	959 (17.5)		
21–30	484 (12.2)	308 (5.7)		
31–40	144 (3.9)	104 (2.0)		
41–50	14 (0.4)	6 (0.1)		

^*∗*^ *p* < 0.05; *n*: unweighted count; %: weighted count; SE: standard error; BMI: body mass index; FPG: fasting plasma glucose; HbA_1c_: glycated hemoglobin; M: mean; DM: diabetes mellitus.

**Table 3:** Comparison of major risk factors for T2DM in Korean adults according to sex (*n* = 9,614).

Variables	Male (*n* = 4,134)	Female (*n* = 5,480)	*t* or *x²*	*p*-value
	*n* (%) or M ± SE	*n* (%) or M ± SE		
Total cholesterol (mg/dL)	198.77 ± 0.73	202.61 ± 0.57	18.89	≤0.001^*∗*^
Low-density lipoprotein cholesterol (mg/dL)	117.73 ± 1.23	125.23 ± 1.88	11.01	≤0.001
High C-reactive protein	1.23 ± 0.03	1.05 ± 0.03	17.19	≤0.001
Systolic BP (mmHg)	121.21 ± 0.31	116.89 ± 0.29	122.15	≤0.001
Diastolic BP (mmHg)	80.77 ± 0.20	75.81 ± 0.17	405.60	≤0.001
Heart rate (/min)	55.96 ± 0.67	56.69 ± 0.59	0.75	0.388
WHtR	0.51 ± 0.00	0.50 ± 0.00	13.75	≤0.001
<0.53	2,312 (66.9)	3,270 (67.5)	0.31	0.580
≥0.53	1,244 (33.1)	1,722 (32.5)		
PA (MET-min/week)	1,151.82 ± 27.18	1,019.86 ± 20.05	17.41	≤0.001
<600	2,241 (51.6)	2,948 (52.1)	11.92	≤0.001
600–1,499	964 (24.7)	1,531 (28.9)		
1,500–2,999	604 (15.1)	687 (12.9)		
≥3,000	325 (8.6)	314 (6.1)		
Sedentary time (h/day)	7.22 ± 0.08	6.92 ± 0.07	10.70	≤0.001
≥8	1,836 (47.6)	2,339 (43.8)	9.90	0.002
<8	2,049 (52.4)	2,917 (56.2)		
Alcohol intake				
None	629 (15.3)	1,756 (31.7)	934.10	≤0.001
1–4 cups/month	1,707 (44.4)	2,943 (56.7)		
≥2 cups/week	1,566 (40.3)	591 (11.6)		
Monthly drinking rate^†^	0.74 ± 0.01	0.43 ± 0.01	733.33	≤0.001
No drinking or <1 cup/month	1,006 (25.7)	3,058 (56.5)	733.33	≤0.001
≥1 cup/month	2,849 (74.3)	2,182 (43.5)		
Sleep time per day (weekly average)	6.54 ± 0.04	6.63 ± 0.03	4.07	0.044
7–8 h	1,723 (45.2)	2,371 (44.7)	0.17	0.684
<7 or >8 h	2,107 (54.8)	2,852 (55.3)		
Hyperlipidemia				
No	3,617 (87.6)	4,631 (85.6)	6.21	0.013
Yes	517 (12.4)	849 (14.4)		
Statin therapy				
No	3,729 (90.7)	4,797 (88.7)	8.13	0.004
Yes	405 (9.3)	683 (11.3)		

^*∗*^ *p* < 0.05; *n*: unweighted count; %: weighted count; SE: standard error; WHtR: waist-to-height ratio; PA: physical activity; MET: metabolic equivalent of task; M: mean; DM: diabetes mellitus. ^†^This variable was calculated by including the total number of participants as the denominator and the number of participants who responded that they had consumed alcohol at least once a month in the last year as the numerator.

**Table 4:** Sex differences in the factors associated with the 4-year estimated incidence of T2DM in Korean adults (*n* = 9,614).

	Male (*n* = 4,134)					Female (*n* = 5,480)				
	B	SE	Β	T	*p*-value	B	SE	*β*	*t*	*p* value
(Constant)	−19.45	1.47		−13.23	≤0.001^*∗*^	−15.62	0.84		−18.59	≤0.001
WHtR	60.15	2.84	0.33	21.19	≤0.001	45.92	1.58	0.38	29.09	≤0.001
Sedentary time	−0.05	0.04	−0.02	−1.31	0.191	0.06	0.03	0.03	2.05	0.041
Monthly drinking rate^†^	1.45	0.31	0.07	4.68	≤0.001	−0.05	0.19	−0.00	−0.27	0.788
Sleep time per day										
1^‡^	−0.58	0.27	−0.03	−2.12	0.034	−0.01	0.19	−0.00	−0.06	0.954
0 (reference)^§^										
	*F* = 120.07, *p* ≤ 0.001, *R*^2^ = 0.113, adj *R*^2^ = 0.112					*F* = 215.15, *p* ≤ 0.001, *R*^2^ = 0.143, adj *R*^2^ = 0.142				

^*∗*^ *p* < 0.05; WHtR: waist-to-height ratio; DM: diabetes mellitus. ^†^This variable was calculated by including the total number of participants as the denominator and the number of participants who responded that they had consumed alcohol at least once a month in the last year as the numerator. ^‡^7–8 h. ^§^<7 h or >8 h.

## Data Availability

The research data used for this study can be accessed from the National Health and Nutrition Data of the Korea Centers for Disease Control and Prevention, available at http://www.kdca.go.kr/yhs/home.jsp through the official procedure.

## References

[1] International Diabetes Federation (2019). *IDF Diabetes Atlas*.

[2] Ministry of Health and Welfare & Korea Disease Control and Prevention Agency (2020). Korea health statistics 2019: Korea national health and nutrition examination survey (KNHANES VIII-1). https://knhanes.cdc.go.kr/knhanes/sub04/sub04_03.do?classType=7.

[3] Korean Diabetes Association (2020). *Diabetes Fact Sheet in Korea 2020*.

[4] Huang Y., Cai X., Mai W., Li M., Hu Y. (2016). Association between prediabetes and risk of cardiovascular disease and all cause mortality: systematic review and meta-analysis. *BMJ*.

[5] Korean Diabetes Association (2019). *2019 Treatment Guideline for Diabetes*.

[6] American Diabetes Association (2020). 2 Classification and diagnosis of diabetes: standards of medical care in diabetes—2020. *Diabetes Care*.

[7] Rådholm K., Chalmers J., Ohkuma T. (2018). Use of the waist-to-height ratio to predict cardiovascular risk in patients with diabetes: results from the ADVANCE-ON study. *Diabetes, Obesity and Metabolism*.

[8] Yun H. S. (2019). Association between diabetes mellitus and drinking behaviors among Koreans adults using data from the 7^th^ (2017) Korean National Health and Nutrition Examination Survey. *AJMAHS*.

[9] Statistics Korea Statistics Research Institute (2019). *Korean Social Trend Report 2019*.

[10] Kautzky-Willer A., Harreiter J., Pacini G. (2016). Sex and gender differences in risk, pathophysiology and complications of type 2 diabetes mellitus. *Endocrine Reviews*.

[11] Britton A., Fat L. N., Neligan A. (2020). The association between alcohol consumption and sleep disorders among older people in the general population. *Scientific Reports*.

[12] Wingard D. L., Berkman L. F. (1983). Mortality risk associated with sleeping patterns among adults. *Sleep*.

[13] Chaput J.-P., Després J.-P., Bouchard C., Astrup A., Tremblay A. (2009). Sleep duration as a risk factor for the development of type 2 diabetes or impaired glucose tolerance: analyses of the Quebec Family Study. *Sleep Medicine*.

[14] Choo K. O., Choi H. H., Park H. J., Kim H. C., Kim I. I. (2015). The relation between sleep duration with diabetes mellitus in Korean adults. *Korean Journal of Family Practice*.

[15] Kennerly A.-M., Kirk A. (2018). Physical activity and sedentary behaviour of adults with type 2 diabetes: a systematic review. *Practical Diabetes*.

[16] Korea Centers for Disease Control and Prevention (2017). *Chronic Disease Current Status and Issues 2017, Chronic Illness factbook [internet]. Cheongju: Korea Centers for Disease Control and Prevention*.

[17] Lim N.-K., Park S.-H., Choi S.-J., Lee K.-S., Park H.-Y. (2012). A risk score for predicting the incidence of type 2 diabetes in a middle-aged Korean cohort. *Circulation Journal*.

[18] World Health Organization *Body Mass Index-BMI*.

[19] Sigmundová D., Sigmund E., Hamřík Z., Kalman M., Pavelka J., Fromel K. (2015). Sedentary behaviour and physical activity of randomised sample of Czech adults aged 20-64 years: IPAQ and GPAQ studies between 2002 and 2011. *Central European Journal of Public Health*.

[20] Arnett D. K, Blumenthal R. S., Albert M. A. (2019). ACC/AHA guideline on the primary prevention of cardiovascular disease: a report of the American college of cardiology/American Heart association task force on clinical practice guidelines. *Journal of the American College of Cardiology*.

[21] Jia A. H., Xu S. Y., Ming J. (2017). The optimal cutoff value of waist-to-height ratio in Chinese: based on cardiovascular risk and metabolic disease. *Zhonghua Nei Ke Za Zhi*.

[22] Fritschi C., Park H., Richardson A. (2016). Association between daily time spent in sedentary behavior and duration of hyperglycemia in type 2 diabetes. *Biological Research For Nursing*.

[23] Korea Disease Control and Prevention Agency *The Seventh Korea National Health and Nutrition Examination Survey (KNHANES VII) (2016–2018) Guidelines. Korea Disease Control and Prevention Agency*.

[24] Lou P., Chen P., Zhang L. (2012). Relation of sleep quality and sleep duration to type 2 diabetes: a population-based cross-sectional survey. *BMJ Open*.

[25] Daoud J. I. (2017). Multicollinearity and regression analysis. *Journal of Physics: Conference Series*.

[26] Feller S., Boeing H., Pischon T. (2010). Body mass index, waist circumference, and the risk of type 2 diabetes mellitus. *Deutsches Aerzteblatt Online*.

[27] Battie C. A., Borja-Hart N., Ancheta I. B., Flores R., Rao G., Palaniappan L. (2016). Comparison of body mass index, waist circumference, and waist to height ratio in the prediction of hypertension and diabetes mellitus: Filipino-American women cardiovascular study. *Preventive Medicine Reports*.

[28] Joseph J. J., Echouffo-Tcheugui J. B., Golden S. H. (2016). Physical activity, sedentary behaviors and the incidence of type 2 diabetes mellitus: the Multi-Ethnic Study of Atherosclerosis (MESA). *BMJ Open Diabetes Research & Care*.

[29] Bakrania K., Edwardson C. L., Khunti K. (2017). Associations of objectively measured moderate-to-vigorous-intensity physical activity and sedentary time with all-cause mortality in a population of adults at high risk of type 2 diabetes mellitus. *Preventive Medicine Reports*.

[30] Holst C., Becker U., Jørgensen M. E., Grønbæk M., Tolstrup J. S. (2017). Alcohol drinking patterns and risk of diabetes: a cohort study of 70,551 men and women from the general Danish population. *Diabetologia*.

[31] Health Insurance Review & Assessment Service (2010). *Stressful Middle-Aged Man, Red Sign of “diabetes*.

[32] He S., Hasler B. P., Chakravorty S. (2019). Alcohol and sleep-related problems. *Current Opinion in Psychology*.

[33] Park S.-Y., Oh M.-K., Lee B.-S. (2015). The effects of alcohol on quality of sleep. *Korean Journal of Family Medicine*.

